# Formation and Control of the Viable but Non-culturable State of Foodborne Pathogen *Escherichia coli* O157:H7

**DOI:** 10.3389/fmicb.2020.01202

**Published:** 2020-06-16

**Authors:** Yanmei Li, Teng-Yi Huang, Congxiu Ye, Ling Chen, Yi Liang, Kan Wang, Junyan Liu

**Affiliations:** ^1^Department of Haematology, Guangzhou Women and Children’s Medical Center, Guangzhou Medical University, Guangzhou, China; ^2^Department of Laboratory Medicine, The Second Affiliated Hospital of Shantou University Medical College, Shantou, China; ^3^Department of Dermato-Venereology, Third Affiliated Hospital of Sun Yat-sen University, Guangzhou, China; ^4^School of Food Science and Engineering, Guangdong Province Key Laboratory for Green Processing of Natural Products and Product Safety, South China University of Technology, Guangzhou, China; ^5^Guangdong Zhongqing Font Biochemical Science and Technology Co. Ltd., Maoming, China; ^6^Research Center for Translational Medicine, The Second Affiliated Hospital, Medical College of Shantou University, Shantou, China; ^7^Department of Civil and Environmental Engineering, A. James Clark School of Engineering, University of Maryland, College Park, MD, United States

**Keywords:** *E. coli* O157:H7, VBNC, food system, control, detection

## Abstract

As a common foodborne pathogen, *Escherichia coli* O157:H7 produces toxins causing serious diseases. However, traditional methods failed in detecting *E. coli* O157:H7 cells in the viable but non-culturable (VBNC) state, which poses a threat to food safety. This study aimed at investigating the formation, control, and detection of the VBNC state of *E. coli* O157:H7. Three factors including medium, salt, and acid concentrations were selected as a single variation. Orthogonal experiments were designed with three factors and four levels, and 16 experimental schemes were used. The formation of the VBNC state was examined by agar plate counting and LIVE/DEAD^®^ BacLight^TM^ bacterial viability kit with fluorescence microscopy. According to the effects of environmental conditions on the formation of the VBNC state of *E. coli* O157:H7, the inhibition on VBNC state formation was investigated. In addition, *E. coli* in the VBNC state in food samples (crystal cake) was detected by propidium monoazide–polymerase chain reaction (PMA-PCR) assays. Acetic acid concentration showed the most impact on VBNC formation of *E. coli* O157:H7, followed by medium and salt concentration. The addition of 1.0% acetic acid could directly kill *E. coli* O157:H7 and eliminate its VBNC formation. In crystal cake, 25, 50, or 100% medium with 1.0% acetic acid could inhibit VBNC state formation and kill *E. coli* O157:H7 within 3 days. The VBNC cell number was reduced by adding 1.0% acetic acid. PMA-PCR assay could be used to detect *E. coli* VBNC cells in crystal cake with detection limit at 10^4^ CFU/ml. The understanding on the inducing and inhibitory conditions for the VBNC state of *E. coli* O157:H7 in a typical food system, as well as the development of an efficient VBNC cell detection method might aid in the control of VBNC *E. coli* O157:H7 cells in the food industry.

## Highlights

– The induction of the VBNC state of *Escherichia coli* by nutritional conditions, acetic acid concentration, and salt concentration was investigated.
– In crystal cake, the VBNC state of *E. coli* could be inhibited by adding 1.0% acetic acid in volume fraction at 4 or −20°C.
– Propidium monoazide–polymerase chain reaction (PMA-PCR) assays could be applied in detection of the VBNC state of *E. coli*.

## Introduction

*Escherichia coli* is one of the major bacterial contaminants associated with foodborne infections worldwide (Zhao et al., 2010b; Sayad et al., 2016; Bao et al., 2017a, b; Xie et al., 2017a; Liu et al., 2018a, b; Renuka et al., 2018; Xu et al., 2020). Since food safety accident caused by *E. coli* was first reported in the United States in 1982, various reports have been posted in China, the United Kingdom, Japan, Australia, and other countries. In recent years, *E. coli* has gradually developed into a health and safety issue of worldwide concern (Xu et al., 2007, 2008a, 2008b, 2011b, 2016a, 2016b; You et al., 2012; Lin et al., 2016; Miao et al., 2016, 2017a, 2017b, 2018; Zhao et al., 2018a, b). The high incidence rate of *E. coli* in food products underlies the urgent need of appropriate strategies for early detection (Zhao et al., 2010b, c, 2011, 2014 2018; Wang et al., 2011; Xu et al., 2011c, 2012b; Lin et al., 2017; Miao et al., 2017c; Liu et al., 2019; Miao et al., 2019). *E. coli* O157:H7, capable of producing Shiga toxin or Shiga-like toxin, can cause hemorrhagic colitis and hemolytic uremic syndrome in human beings (Neil et al., 2012). Shiga toxin causes various diseases including bloody diarrhea and severe hemolytic uremic syndrome, leading to kidney failure. *E. coli* O157:H7 is a typical foodborne pathogen, colonizing in food systems and drinking water (Ercumen et al., 2017; Bourely et al., 2018; Zeinhom et al., 2018).

The standard detection method for foodborne pathogens is agar plate counting (Xu et al., 2008a, b, 2009; 2010; 2011a; 2012a; 2016c; 2017a; 2017b; 2018; Zhong et al., 2013; Zhang et al., 2015; Bao et al., 2017c; Xie et al., 2017b; Jia et al., 2018; Wen et al., 2020). However, this method fails in detecting viable but non-culturable (VBNC) state cells that were first reported in 1982 (Xu et al., 1982). Up to now, 85 types of microorganisms have been found to enter the VBNC state, including 18 non-pathogenic bacteria and 67 pathogenic bacteria (Ramamurthy et al., 2014). Studies have shown that *E. coli* has the ability to enter the VBNC state (Yaron and Matthews, 2002; Zhang et al., 2015; Liu et al., 2017b; Afari et al., 2019). VBNC cells differ from normal cells in both physiology and morphology. However, VBNC cells acquire high ATP concentrations (Lindback et al., 2010) and metabolic activity based on the expression of certain genes (Yaron and Matthews, 2002).

Bacteria in the VBNC state fail to form colonies in routine detection agar plates under certain environmental stresses. However, these cells have food spoilage and pathogenic capacity (Liu et al., 2017b). Factors including adverse nutrition levels, temperatures, and osmotic pressures, which are frequently encountered during food processing and storage environments, have been reported to induce bacteria entering the VBNC state. With the failure in detection by routine method and the capability in causing food safety problems, the VBNC state of *E. coli* O157:H7 is a major concern in the food industry (Liu et al., 2017b).

Crystal cake is a traditional Chinese snack that has the characteristics of rich nutrition. Its rich nutrients and sufficient water make it a natural medium for various pathogenic bacteria and spoilage bacteria, including *E. coli*. In this study, crystal cake was applied as a representative food system to study the formation, control, and detection of the VBNC state of *E. coli* O157:H7 under food processing and storage conditions.

## Materials and Methods

### Cultivation of *E. coli* O157:H7

*Escherichia coli* O157:H7 ATCC25922 was stored at -80°C in Luria-Bertani (LB) broth containing 20% (v/v) glycerol. It was streaked on LB agar plate and grown at 37°C for 24 h. Then, a single colony was inoculated into 2 ml of LB broth and incubated at 37°C for 12 h using a shaker incubator set at 150 r/min. The bacteria suspension was diluted 1:100 in fresh medium and cultured for 4 h to the log phase upon further experiments.

### Induction of the VBNC State

According to the optimal culture conditions of *E. coli* O157:H7, and the principle of reverse adjustment, conditions that are unfavorable to bacterial growth were selected as candidate factors to induce the VBNC state. Three factors including nutritional state, salt concentration, and acidity were taken as a single variable. To investigate possible VBNC induction conditions, three factors and four levels of orthogonal experiments were designed, and 16 protocols (Table 1) were applied. Log phase cells were centrifuged for 10 min at 5000 r/min and the pellet was washed once with 1 × phosphate buffer solution and then resuspended in the induced solution. The initial concentration of the bacterial solution was diluted to approximately 10^7^ CFU/ml. To avoid the effects of repeated freezing and thawing, the bacterial suspension under each condition was mixed and divided into multiple 1.5-ml centrifuge tubes, followed by induction in refrigerators at 4 and −20°C, respectively.

**TABLE 1:** The experimental methods of orthogonal array design of VBNC induction of *E. coli* O157:H7.

**Protocol number**	**LB medium concentration (%)**	**NaCl concentration (m/v) (%)**	**Acetic acid concentration (v/v) (%)**
1	0	0.9	0
2	25	0.9	0.3
3	50	0.9	0.7
4	100	0.9	1
5	25	5	0
6	0	5	0.3
7	100	5	0.7
8	50	5	1
9	50	10	0
10	100	10	0.3
11	0	10	0.7
12	25	10	1
13	100	15	0
14	50	15	0.3
15	25	15	0.7
16	0	15	1

### Determination of Culturable Cell Number During Storage

The culturable cell number in each of the VBNC inducing bacterial culture was determined every 3 days by a plate counting method. Briefly, the culture of *E. coli* O157:H7 was serial diluted in 0.9% NaCl, spread on LB agar plates, and incubated at 37°C for 24 h. When the culturable number is 1 CFU/ml, the cells were considered non-culturable and possibly enter the VBNC state (Piao et al., 2019).

### Determination of Viable Cell Number

To determine if the non-culturable cells are in the VBNC state after exposure to the respective treatment conditions, the LIVE/DEAD^®^ BacLight^TM^ bacterial viability kit (Thermo Fisher Scientific, China) was used. Five hundred microliters of the non-culturable bacterial cell sample was centrifuged at 5000 r/min for 15 min, washed with saline twice, and resuspended in saline. Subsequently, 1.5 μl of SYTO 9 dye and 1.5 μl of propidium iodide were added to the sample, followed by mixing and 30 min incubation in the dark. After incubation, 5-μl cells were captured between a slide and a coverslip and used for fluorescence microscopy. The appearance of green cells indicates the existence of VBNC cells.

### Inhibition of VBNC State by Acidity and Nutritional Conditions

According to the VBNC state induction process, appropriate conditions were selected to inhibit the formation of the VBNC state. The configured medium was resuspended, and the initial concentration of *E. coli* O157:H7 was adjusted to 10^7^ CFU/ml. The bacterial solution with a volume of 30 ml was stored at 4 and −20°C, respectively. The number of cultivable bacteria was measured by plate counting every 3 days (Tables 2, 3).

**TABLE 2:** Inhibition assay of acidity on the formation of VBNC state of *E. coli* O157:H7.

**Protocol number**	**LB medium concentration (%)**	**NaCl concentration (m/v) (%)**	**Acetic acid concentration (v/v) (%)**
1	100	10	0.7
2			1
3	100	15	0.7
4			1
5	50	15	0.7
6			1

**TABLE 3:** Inhibition assay of nutritional status on the formation of VBNC state of *E. coli* O157:H7.

**Protocol number**	**LB medium concentration (%)**	**NaCl concentration (m/v) (%)**	**Acetic acid concentration (v/v) (%)**
1	0	10	0.3
2	25		
3	0	15	0
4	25		
5	0	15	0.3
6	25		

### Elimination of the VBNC State in Crystal Cake

According to the National Food Safety standards (GB4789.3-2016) in China, 25 g of crystal cake was added to 225 ml of saline, and the concentration of this medium was determined to be 100, 25, and 50% of the food sample culture medium and is configured by dilution and used after autoclaving. Subsequently, *E. coli* O157:H7 was cultured to the logarithmic phase and centrifuged at 4°C, the supernatant was discarded, and the suspension was resuspended three times with physiological saline. The bacterial cells were resuspended using the sterilized food sample medium, and the concentrations of *E. coli* O157:H7 were adjusted to 10^7^ CFU/ml, respectively, and a sterile acetic acid solution was added to fix the volume fraction of acetic acid to 1.0%. The induction solution was stored at 4°C, and the culturable number and activity were detected by a plate method combined with a LIVE/DEAD^®^ BacLight^TM^ bacterial viability kit.

### PMA-PCR Assays

Twenty-five grams of crystal cake was mixed with 225 ml of physiological saline and sterilized, and *E. coli* in the VBNC state was added. Bacterial suspensions with initial concentrations of 10^6^, 10^5^, 10^4^, 10^3^, 10^2^, and 10 CFU/ml were obtained. Five hundred microliters of the bacteria suspension was added to a 1.5-ml centrifuge tube, and the PMA working solution was added to a final concentration of 5 μg/ml (to distinguish between the VBNC state and dead cells). After mixing, the samples were kept at room temperature for 10 min in the dark. Subsequently, the centrifuge tube was placed on ice, and 15 cm away from a 650 W halogen lamp for 5 min to complete the binding of PMA and DNA. The PMA–DNA was centrifuged at 10,000 r/min for 5 min, and the supernatant was discarded. DNA was then extracted using a bacterial group DNA extraction kit (Dongsheng Biotech, Guangzhou). The extracted DNA was detected by PCR. PCR assay was performed in a 25-μl volume and with 0.6 μM primers (*rfbE*-F: TTGGCATCGTGTGGACAGGGTAGGACCGCAGAGGAAAG A; *rfbE*-R: TGGGACAGGTGTGCTACGGTTTCCACGCC AACCAAGATC). The thermal profile for PCR mixtures was 94°C for 5 min, followed by 30 cycles of 94°C for 30 s, 52°C for 60 s, and 72°C for 90 s and a final extension cycle at 72°C for 7 min. The amplified products (5 μl/well) were analyzed by gel electrophoresis in 2% agarose gels and stained with ethidium bromide for 10 min. A negative control was included using sterile water instead of culture or DNA template.

## Results and Discussion

### Formation of the VBNC State

**FIGURE 1:**
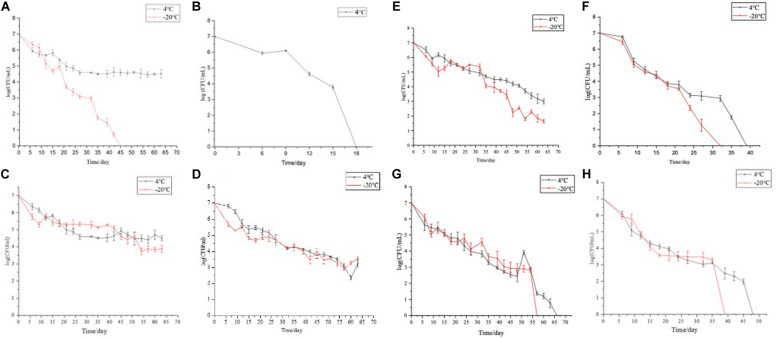
The culturable number of *E. coli* O157:H7 stored under 16 different conditions (4 and −20°C). **(A–H)** The cell viability of *E. coli* O157:H7 when stored at 4 and −20°C under protocols 2, 10, 13, and 14, respectively.

To induce the formation of the VBNC state, 16 induction solutions were applied (Table 1). In induction solution 1, when stored at 4°C, the culturable cell number of *E. coli* O157:H7 was reduced to 5 × 10^4^ CFU/ml in 66 days. When stored at −20°C, the culturable cell number of *E. coli* O157:H7 dropped to 0 in 3 days (Figure 1A). In induction solution 2, the number of culturable cells reduced to 0 after 18 days of storage at 4°C, and the number of culturable cells reduced to 0 within 3 days of storage at −20°C (Figure 1B). In induction solution 10, the number of culturable cells reduced to 0 after storing at 4 and −20°C for 39 and 33 days, respectively (Figure 1F). In induction solution 13, the culturable cell number reduced to 0 in 48 and 39 days of storage at 4 and −20°C, respectively (Figure 1G). In induction solution 14, the culturable cell number reduced to 0 in 57 and 66 days when stored at 4 and −20°C, respectively (Figure 1H). In induction solution 5, 7, and 9, although with a downtrend within 66 days, the number of culturable cells did not decrease to 0 (Figures 1C–E). In addition, in induction solutions 3, 4, 6, 8, 11, 12, 15, and 16, the number of culturable cells of *E. coli* O157:H7 reduced to 0 in 3 days.

**FIGURE 2:**
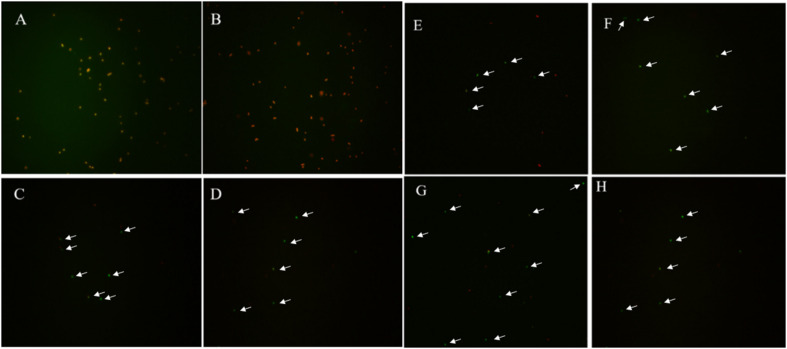
The viability of un-culturable *E. coli* O157:H7 stored at different conditions with fluorescence. **(A–H)** *E. coli* O157:H7 inoculated in the medium configured according to the method 2, 10, 13, 14, and stored at 4 or −20°C, respectively.

For the induction solutions that were capable of inducing the culturable cell number of *E. coli* O157:H7 to 0 (Table 4), the existence of viable cells was determined. When the *E. coli* O157:H7 culture in induction solution 2 was stored at 4°C for 18 days, the cells were all dead, indicating that *E. coli* O157:H7 did not enter the VBNC state (Figure 2). The fluorescence results of *E. coli* O157:H7 in induction solutions 1, 10, 13, and 14 showed that both dead and viable cells were detected, indicating that *E. coli* O157:H7 entered the VBNC state under these conditions.

**TABLE 4:** The time of culturable number of *E. coli* O157:H7 decreased to 0 stored at different methods.

**Protocol number**	**4°C**	**−20°C**	**Protocol number**	**4°C**	**−20°C**
1	+	45 days	9	+	+
2	18 days	/	10	39 days	33 days
3	/	/	11	/	/
4	/	/	12	/	/
5	+	+	13	48 days	39 days
6	/	/	14	57 days	66 days
7	+	+	15	/	/
8	/	/	16	/	/

*“+” stands for *E. coli* is culturable and “/” stands for the number of cultivable *E. coli* in 3 days has dropped to 0.*

### Factors Affecting the Survival of *E. coli* O157:H7 Cells

In the 16 induction solutions, factors including salt, acetic acid, and medium concentrations were applied and analyzed on the effect on the survival of *E. coli* O157:H7 cells. First, the effect of salt and acid concentrations on the survival of *E. coli* O157:H7 cells was investigated. In induction solutions 3, 8, 9, and 14, the medium concentration is 50%. In induction solutions 3 (acid: 0.7%, salt: 0.9%) and 8 (acid: 1%, salt: 5%), the culturable cell number of *E. coli* O157:H7 reduced to 0 in 3 days. However, in induction solutions 9 (acid: 0, salt: 10%) and 14 (acid: 0.3%, salt: 15%), *E. coli* O157:H7 cells survived for more than 57 days. Especially in induction solution 9, the *E. coli* O157:H7 cells survived for more than 66 days. In induction solutions 4, 10, and 13, the medium concentration is 100%. In induction solution 4 (acid: 1%, salt: 0.9%), *E. coli* O157:H7 all died in 3 days and did not enter the VBNC state. In induction solutions 10 (acid: 0.3%, salt: 10%) and 13 (acid: 0, salt: 15%), *E. coli* O157:H7 cells were able to survive for 33–48 days. These results suggest that the effect of acetic acid concentration on the survival of *E. coli* O157:H7 is stronger than that of salt concentration.

Second, the effect of acid and medium conditions on the survival of *E. coli* O157:H7 cells under a certain salt concentration were investigated. In induction solutions 1, 2, 3, and 4, the salt concentration is 0.9%. In induction solutions 1 (medium: 0, acid: 0) and 2 (medium: 25%, acid: 0.3%), *E. coli* O157:H7 cells survived for more than 66 and 18 days at 4°C, respectively. However, in induction solutions 3 (medium: 50%, acid: 0.7%) and 4 (medium: 100%, acid: 1%), the *E. coli* O157:H7 cells all died in 3 days. It showed the effect of acid on the survival of *E. coli* O157:H7 is stronger than that of nutrients. In addition, when the acid concentration is 1% in induction solutions 4, 8, 12, and 16, *E. coli* O157:H7 cells died in 3 days regardless of the medium and salt concentrations. Especially in induction solution 4 (medium: 100%, acid: 0.9%) with the premium centration of medium and acid, *E. coli* O157:H7 cells still died in 3 days, indicating that acidity has the most significant effect on the survival of *E. coli* O157:H7 cells.

Third, under the same acetic acid concentration, the effects of salt and medium concentrations on the survival of *E. coli* O157:H7 were investigated. In induction solutions 6, 10, and 14, the acetic acid concentration is 0.3%. In induction solution 6 (medium: 0, salt: 5%), *E. coli* O157:H7 cells died within 3 days. However, in induction solutions 10 (medium: 100%, salt: 10%) and 14 (medium: 50%, salt: 15%), although the salt concentration increased, *E. coli* O157:H7 cells were able to survive from 33 to 66 days due to the high medium concentration. It indicated that the increase in the medium concentration eliminated the adverse effect of the increase in salt concentration. Among the factors on the survival of *E. coli* O157:H7, the effect of the medium concentration might be greater than that of the salt concentration.

When the environment changes, porin is crucial for the survival of *E. coli*. The major external protein omp (ompF, ompC) of *E. coli* is regulated by *envZ* (osmotic pressure sensor protein) and *ompR*. Studies have showed that wild-type, *ompF*, and *ompC* mutant strain of *E. coli* can enter the VBNC state under environmental pressure (pH, osmotic pressure, and starvation stress conditions), but in the *envZ* mutant strain, they cannot enter the VBNC state. This shows that the *envZ* mutant strains cannot sense changes in the external environment. When the strains are exposed to adverse conditions, they cannot enter the VBNC state (Pienaar et al., 2016). The expression of (p) ppGpp (guanosine pentaphosphate or guanosine tetraphosphate) synthetic genes *relA* and *spoT* are identified to upregulate in the VBNC state of *E. coli* O157:H7 (Magnusson et al., 2005; Mishra et al., 2012). Compared with normal strains, mutants that failed to synthesize (p) ppGpp lost culturability more quickly, and their ability to enter the VBNC state was significantly reduced. When (p) ppGpp was overexpressed, the number of VBNC cells was significantly increased (Boaretti et al., 2003). When bacteria suffered from amino acid starvation, the expression of *RelA* or *SpoT* increased. (p) ppGpp synthesis increased and degradation decreased. In addition, exopolyphosphatase (PPX) was degraded by (p) ppGpp of polyphosphates (Ayrapetyan et al., 2018).

### Effects of Acidity and Nutrition on Formation of the VBNC State

Among the factors affecting the survival of *E. coli* O157:H7, acetic acid concentration plays a major role, followed by medium and salt concentrations. Considering the actual situation in the food system and in the process of food processing, exploring the concentration of acetic acid and culture medium to control the normal state of *E. coli* O157:H7 and VBNC is meaningful.

When the acetic acid concentration is 1%, the *E. coli* O157:H7 cells died within 3 days in induction solutions 3 (medium: 100%, salt: 10%), 4 (medium: 100%, salt: 15%), and 6 (medium: 50%, salt: 15%). When the acetic acid concentration is 0.7%, *E. coli* O157:H7 could survive in induction solutions 1 (medium: 100%, salt: 10%) and 3 (medium: 100%, salt: 15%). In induction solution 5 (medium: 50%, salt: 15%), *E. coli* O157:H7 cells died within 3 days. When the medium concentration is higher than 50%, treatment with 1.0% acetic acid directly killed *E. coli* O157:H7 without entering the VBNC state (Table 5).

**TABLE 5:** Inhibition of acidity on the formation of VBNC state of *E. coli* O157:H7.

**Protocol number**	**Culturable**		**Viable**	
	**4°C**	**−20°C**	**4°C**	**−20°C**
1	+	+	ND	ND
2	/	/	−	−
3	+	+	ND	ND
4	/	/	−	−
5	/	/	−	−
6	/	/	−	−

*“+” stands for *E. coli* is culturable, “/” stands for *E. coli* is not culturable, “-” stands for *E. coli* is not active, and “ND” stands for *E. coli* activity not detected.*

When the medium concentration is 25%, *E. coli* O157:H7 cells survived more than 3 days in induction solutions 2 (salt: 10%, acid: 0.3%), 4 (salt: 15%, acid: 0), and 6 (salt: 15%, acid: 0.3). When the medium concentration is 0, in induction solutions 1 (salt: 10%, acid: 0.3%) and 5 (salt: 15%, acid: 0.3%), the number of culturable and viable cells was both 0 in 3 days. In induction solution 3 (salt: 15%, acid: 0), *E. coli* O157:H7 cells were able to survive for more than 3 days. Under weak acid and high salt conditions, the formation of the VBNC state of *E. coli* O157:H7 was controlled by changing the nutritional condition (Table 6).

**TABLE 6:** Inhibition of nutritional status on the formation of VBNC state of *E. coli* O157:H7.

**Protocol number**	**Culturable**		**Viable**	
	**4°C**	**−20°C**	**4°C**	**−20°C**
1	/	/	−	−
2	+	+	ND	ND
3	+	+	ND	ND
4	+	+	ND	ND
5	/	/	−	−
6	+	+	ND	ND

*“+” stands for *E. coli* is culturable, “/” stands for *E. coli* is not culturable, “-” stands for *E. coli* is not active, and “ND” stands for *E. coli* activity not detected.*

In induction solutions 2, 3, 4, and 6, the culturable cell number of *E. coli* O157:H7 did not decrease to 0 in 3 days. However, the culturable cell numbers of *E. coli* O157:H7 reduced to 0 after storing at 4 and −20°C for 3 days in induction solutions 1 and 5, and the cells were not viable. Thus, changing the acid concentration with cold storage might be efficient to control the formation of the VBNC state of *E. coli* O157:H7.

### Elimination of the VBNC State in Crystal Cake

**FIGURE 3:**
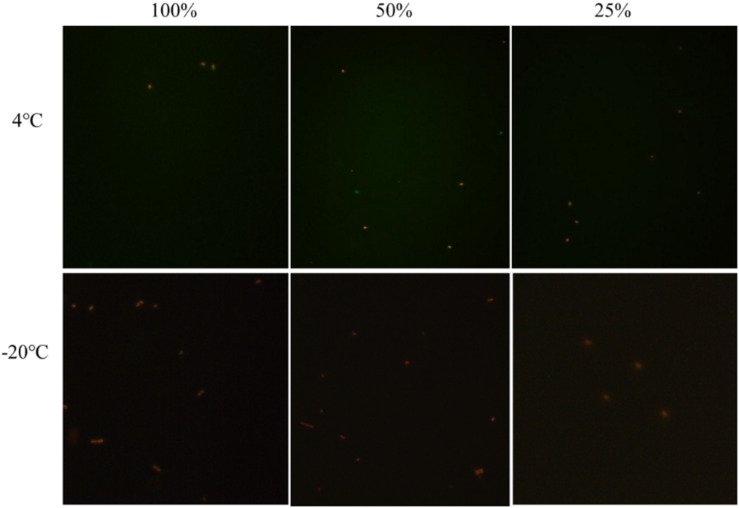
The viability of un-culturable *E. coli* O157:H7 stored at different conditions with fluorescence.

In the crystal cake food system with 100, 50, and 25% nutrient concentrations and 1.0% acetic acid, the culturable cell numbers of *E. coli* O157:H7 were 0 after storing at 4 and −20°C for 3 days. The results of fluorescence microscopy showed that all the *E. coli* O157:H7 cells died, suggesting *E. coli* O157:H7 was not capable of entering into the VBNC state under these conditions (Figure 3). In the food system, without affecting the flavor and quality of the food, the normal and the VBNC state of *E. coli* O157:H7 was controlled and eliminated by adding 1% acetic acid.

The foodborne microorganisms in the VBNC state have food spoilage ability. Compared with normal state, VBNC state cells have reduced metabolic activity and increased resistance of the cell wall to external environmental stress, including high concentrations of antibiotics, heavy metal ions, high temperature, high salt, and higher acidity (Ayrapetyan and Oliver, 2016; Shekar et al., 2017; Schottroff et al., 2018). Due to the presence of resistance, *E. coli* O157:H7 in the VBNC state is more difficult to be completely eliminated. Under favorable conditions, *E. coli* O157:H7 in the VBNC state might be able to overcome this inactive state and become active again, thus being able to reproduce in the food system. During the processing of crystal cake, the surface of the processing equipment and the inside of the pipeline should be cleaned in time to ensure no residual nutrients, thereby inhibiting the survival of *E. coli* O157:H7. In addition, combined with a certain concentration of acetic acid treatment equipment surface (1.0% acetic acid), it ensures the elimination of *E. coli* O157:H7 and its VBNC state and avoid potential food safety risks.

### Detection of the VBNC State of *E. coli* in Crystal Cake

The risk of food safety issues can be partially reduced by the efficient detection of the VBNC state of *E. coli* in food system. Propidium monoazide (PMA) is a photoreactive DNA-binding dye. Dead microorganisms lose their capability to keep their membranes intact, which leaves the “naked” DNA in the cytosol ready to react with PMA. DNA of living organisms are not exposed to the PMA, as they have a complete cell membrane. DNA extraction and PCR amplification of PMA-treated samples can effectively detect bacteria in the VBNC state. The detection limit was determined by detecting different concentrations of *E. coli* O157:H7 in the VBNC state. In this study, the detection limit of the VBNC state *E. coli* O157 in the crystal cake food system using PMA–polymerase chain reaction (PMA-PCR) was 10^4^ CFU/ml, which can effectively avoid the potential threat brought by VBNC state bacteria.

## Conclusion

The effect of external environmental conditions on the formation of the VBNC state of *E. coli* O157:H7 was as follows: acidity > nutritional state > salt concentration. When the nutrient concentration is higher than 50%, *E. coli* O157:H7 was killed by adding 1.0% acetic acid. In the crystal cake system, when the nutrient concentration is 25, 50, and 100%, *E. coli* O157:H7 cells died in 3 days by adding 1.0% acetic acid with no VBNC cells identified. In the food system, the VBNC state formation of *E. coli* O157:H7 was inhibited by adding 1.0% acetic acid. In addition, PMA-PCR assays can be utilized in the detection of the VBNC state of *E. coli* in the food system.

## Data Availability Statement

All datasets presented in this study are included in the article/supplementary material.

## Author Contributions

JL and KW conceived the study and participated in its design and coordination. YML and T-YH performed the experimental work. CY and LC analyzed the data. YL and JL prepared and revised this manuscript. All authors reviewed and approved the final manuscript.

## Conflict of Interest

The author YL was employed by Guangdong Zhongqing Font Biochemical Science and Technology Co. Ltd. The remaining authors declare that the research was conducted in the absence of any commercial or financial relationships that could be construed as a potential conflict of interest.

## References

[B1] Afari G. K. Liu H. Hung Y. (2019). The effect of produce washing using electrolyzed water on the induction of the viable but non-culturable (VBNC) state in *Listeria monocytogenes* and *Escherichia coli* O157:H7. *LWT Food Sci. Technol.* 110 275–282. 10.1016/j.lwt.2019.04.089

[B2] Ayrapetyan M. Oliver J. D. (2016). The viable but non-culturable state and its relevance in food safety. *Curr. Opin. Food Sci.* 8 127–133. 10.1016/j.cofs.2016.04.010

[B3] Ayrapetyan M. Williams T. Oliver J. D. (2018). Relationship between the viable but nonculturable state and antibiotic persister cells. *J. Bacteriol.* 200:e0249-18. 10.1128/JB.00249-18 PMC6153661 30082460

[B4] Bao X. Jia X. Chen L. Peters B. M. Lin C. Chen D. (2017a). Effect of polymyxin resistance (pmr) on biofilm formation of *Cronobacter sakazakii*. *Microb. Pathogen.* 106 16–19. 10.1016/j.micpath.2016.12.012 28012985

[B5] Bao X. Yang L. Chen L. Li B. Li L. Li Y. (2017b). Analysis on pathogenic and virulent characteristics of the *Cronobacter sakazakii* strain BAA-894 by whole genome sequencing and its demonstration in basic biology science. *Microb. Pathogen.* 109 280–286. 10.1016/j.micpath.2017.05.030 28546117

[B6] Bao X. Yang L. Chen L. Li B. Li L. Li Y. (2017c). Virulent and pathogenic features on the *Cronobacter sakazakii* polymyxin resistant pmr mutant strain s-3. *Microb. Pathogen.* 110 359–364. 10.1016/j.micpath.2017.07.022 28711508

[B7] Boaretti M. Lleo M. M. Bonato B. Signoretto C. Canepari P. (2003). Involvement of rpoS in the survival of *Escherichia coli* in the viable but non-culturable state. *Environ. Microbiol.* 5 986–996. 10.1046/j.1462-2920.2003.00497.x 14510852

[B8] Bourely C. Chauvin C. Jouy E. Cazeau G. Jarrige N. Leblond A. (2018). Comparative epidemiology of *E. coli* resistance to third-generation cephalosporins in diseased food-producing animals. *Vet. Microbiol.* 223 72–78. 10.1016/j.vetmic.2018.07.025 30173755

[B9] Ercumen A. Pickering A. J. Kwong L. H. Arnold B. F. Parvez S. M. Alam M. (2017). Animal feces contribute to domestic fecal contamination: evidence from *E-coli* measured in water, hands, food, flies, and soil in Bangladesh. *Environ. Sci. Technol.* 51 8725–8734. 10.1021/acs.est.7b01710 28686435 PMC5541329

[B10] Jia X. Hua J. Liu L. Xu Z. Li Y. (2018). Phenotypic characterization of pathogenic *Cronobacter* spp. strains. *Microb. Pathogen.* 121 232–237. 10.1016/j.micpath.2018.05.033 29800699

[B11] Lin S. Li L. Li B. Zhao X. Lin C. Deng Y. (2016). Development and evaluation of quantitative detection of n-epsilon-carboxymethyl-lysine in *Staphylococcus aureus* biofilm by LC-MS method. *Basic Clin. Pharmacol.* 118 33–33.

[B12] Lin S. Yang L. Chen G. Li B. Chen D. Li L. (2017). Pathogenic features and characteristics of food borne pathogens biofilm: biomass, viability and matrix. *Microb. Pathogen.* 111 285–291. 10.1016/j.micpath.2017.08.005 28803003

[B13] Lindback T. Rottenberg M. E. Roche S. M. Rorvik L. M. (2010). The ability to enter into an avirulent viable but non-culturable (VBNC) form is widespread among *Listeria monocytogenes* isolates from salmon, patients and environment. *Vet. Res.* 41:8. 10.1051/vetres/2009056 PMC2775167 19796607

[B14] Liu J. Li L. Li B. Peters B. M. Deng Y. Xu Z. (2017a). Study on spoilage capability and VBNC state formation and recovery of *Lactobacillus plantarum*. *Microb. Pathogen.* 110 257–261. 10.1016/j.micpath.2017.06.044 28668605

[B15] Liu J. Zhou R. Li L. Peters B. M. Li B. Lin C. (2017b). Viable but non-culturable state and toxin gene expression of enterohemorrhagic *Escherichia coli* 0157 under cryopreservation. *Res. Microbiol.* 168 188–193. 10.1016/j.resmic.2016.11.002 27884785

[B16] Liu L. Lu Z. Li L. Li B. Zhang X. Xu Z. (2018a). Physical relation and mechanism of ultrasonic bactericidal activity on pathogenic *E. coli* with WPI. *Microb. Pathogen.* 117 73–79. 10.1016/j.micpath.2018.02.007 29428425

[B17] Liu L. Xu R. Li L. Li B. Zhang X. Xu Z. (2018b). Correlation and in vitro mechanism of bactericidal activity on E. coli with whey protein isolate during ultrasonic treatment. *Microb. Pathogen.* 115 154–158. 10.1016/j.micpath.2017.12.062 29278782

[B18] Liu L. Ye C. Soteyome T. Zhao X. Xia J. Xu W. (2019). Inhibitory effects of two types of food additives on biofilm formation by foodborne pathogens. *Microbiologyopen* 8:e00853. 10.1002/mbo3.853 PMC6741122 31179649

[B19] Magnusson L. U. Farewell A. Nystrom T. (2005). ppGpp: a global regulator in *Escherichia coli*. *Trends. Microbiol.* 13 236–242. 10.1016/j.tim.2005.03.008 15866041

[B20] Miao J. Chen L. Wang J. Wang W. Chen D. Li L. (2017a). Current methodologies on genotyping for nosocomial pathogen methicillin-resistant *Staphylococcus aureus* (MRSA). *Microb. Pathogen.* 107 17–28. 10.1016/j.micpath.2017.03.010 28284852

[B21] Miao J. Chen L. Wang J. Wang W. Chen D. Li L. (2017b). Evaluation and application of molecular genotyping on nosocomial pathogen-methicillin-resistant *Staphylococcus aureus* isolates in Guangzhou representative of Southern China. *Microb. Pathogen.* 107 397–403. 10.1016/j.micpath.2017.04.016 28414166

[B22] Miao J. Liang Y. Chen L. Wang W. Wang J. Li B. (2017c). Formation and development of *Staphylococcus* biofilm: with focus on food safety. *J. Food Saf.* 7:e012358.

[B23] Miao J. Lin S. Soteyome T. Peters B. M. Li Y. Chen H. (2019). Biofilm formation of *Staphylococcus aureus* under food heat processing conditions: first report on cml production within biofilm. *Sci. Rep.* 9:1331. 10.1038/s41598-018-35558-2 PMC6361893 30718527

[B24] Miao J. Peters B. M. Li L. Li B. Zhao X. Xu Z. (2016). Evaluation of ERIC-PCR for fingerprinting methicillin-resistant *Staphylococcus aureus* strains. *Basic Clin. Pharmacol.* 118 33–33.

[B25] Miao J. Wang W. Xu W. Su J. Li L. Li B. (2018). The fingerprint mapping and genotyping systems application on methicillin-resistant *Staphylococcus aureus*. *Microb. Pathogen.* 125 246–251. 10.1016/j.micpath.2018.09.031 30243550

[B26] Mishra A. Taneja N. Sharma M. (2012). Viability kinetics, induction, resuscitation and quantitative real-time polymerase chain reaction analyses of viable but nonculturable *Vibrio cholerae* O1 in freshwater microcosm. *J. Appl. Microbiol.* 112 945–953. 10.1111/j.1365-2672.2012.05255.x 22324483

[B27] Neil K. P. Biggerstaff G. MacDonald J. K. Trees E. Medus C. Musser K. A. (2012). A novel vehicle for transmission of *Escherichia coli* O157:H7 to humans: multistate outbreak of *E. coli* O157:H7 infections associated with consumption of ready-to-bake commercial prepackaged cookie dough–United States, 2009. *Clin. Infect. Dis.* 54 511–518. 10.1093/cid/cir831 22157169

[B28] Piao M. Li Y. Wang Y. Wang F. Zhen T. Deng Y. (2019). Induction of viable but putatively non-culturable *Lactobacillus acetotolerans* by thermosonication and its. *LWT Food Sci. Technol.* 109 313–318. 10.1016/j.lwt.2019.04.046

[B29] Pienaar J. A. Singh A. Barnard T. G. (2016). The viable but non-culturable state in pathogenic *Escherichia coli*: a general review. *Sci. Direct.* 5:e00368. 10.4102/ajlm.v5i1.368 PMC5436400 28879110

[B30] Ramamurthy T. Ghosh A. Pazhani G. P. Shinoda S. (2014). Current perspectives on viable but non-culturable (VBNC) pathogenic bacteria. *Front. Public Health* 2:103. 10.3389/fpubh.2014.00103 25133139 PMC4116801

[B31] Renuka R. M. Achuth J. Chandan H. R. Venkataramana M. Kadirvelu K. (2018). A fluorescent dual aptasensor for the rapid and sensitive onsite detection of E-coli O157:H7 and its validation in various food matrices. *New J. Chem.* 42 10807–10817. 10.1039/c8nj00997j

[B32] Sayad A. A. Ibrahim F. Uddin S. M. Pei K. X. Mohktar M. S. Madou M. (2016). A microfluidic lab-on-a-disc integrated loop mediated isothermal amplification for foodborne pathogen detection. *Sens. Actua. B Chem.* 227 600–609. 10.1016/j.snb.2015.10.116

[B33] Schottroff F. Froehling A. Zunabovic P. M. Krottenthaler A. Schlueter O. (2018). Sublethal injury and viable but non-culturable (VBNC) state in microorganisms during preservation of food and biological materials by non-thermal processes. *Front. Microbiol.* 9:2773 10.3389/fpubh.2014.02773 PMC6255932 30515140

[B34] Shekar A. Babu L. Ramlal S. Sripathy M. H. Batra H. (2017). Selective and concurrent detection of viable *Salmonella* spp., *E-coli*, *Staphylococcus aureus*, *E-coli* 0157:H7, and *Shigella* spp., in low moisture food products by PMA-mPCR assay with internal amplification control. *LWT Food Sci. Technol.* 86 586–593. 10.1016/j.lwt.2017.08.023

[B35] Wang L. Zhao X. Chu J. Li Y. Li Y. Li C. (2011). Application of an improved loop-mediated isothermal amplification detection of Vibrio parahaemolyticus from various seafood samples. *AFR J. Microbiol. Res.* 5 5765–5771.

[B36] Wen S. Feng D. Chen D. Yang L. Xu Z. (2020). Molecular epidemiology and evolution of *Haemophilus influenzae*. *Infect. Genet. Evol.* 80:104205. 10.1016/j.meegid.2020.104205 31981610

[B37] Xie J. Peters B. M. Li B. Li L. Yu G. Xu Z. (2017a). Clinical features and antimicrobial resistance profiles of important *Enterobacteriaceae* pathogens in Guangzhou representative of Southern China, 2001-2015. *Microb. Pathogen.* 107 206–211. 10.1016/j.micpath.2017.03.038 28365324

[B38] Xie J. Yang L. Peters B. M. Chen L. Chen D. (2017b). A 16-year retrospective surveillance report on the pathogenic features and antimicrobial susceptibility of *Pseudomonas aeruginosa* isolates from FAHJU in Guangzhou representative of Southern China. *Microb. Pathogen.* 110 37–41. 10.1016/j.micpath.2017.06.018 28629721

[B39] Xu H. S. Roberts N. Singleton F. L. Attwell R. W. Grimes D. J. Colwel R. R. (1982). Survival and viability of nonculturable *Escherichia coli* and *Vibrio cholerae* in the estuarine and marine environment. *Microb. Ecol.* 8 313–323. 24226049 10.1007/BF02010671

[B40] Xu Z. Gui Z. Zhao X. Zhang Y. He X. Li W. (2012a). Expression and purification of gp41-gp36 fusion protein and application in serological screening assay of HIV-1 and HIV-2. *AFR J. Microbiol. Res.* 6 6295–6299.

[B41] Xu Z. Li L. Chu J. Peters B. M. Harris M. L. Li B. (2012b). Development and application of loop-mediated isothermal amplification assays on rapid detection of various types of staphylococci strains. *Food Res. Int.* 47 166–173. 10.1016/j.foodres.2011.04.042 22778501 PMC3390935

[B42] Xu Z. Hou Y. Peters B. M. Chen D. Li B. Li L. (2016a). Chromogenic media for MRSA diagnostics. *Mol. Biol. Rep.* 43 1205–1212. 10.1007/s11033-016-4062-3 27562853

[B43] Xu Z. Hou Y. Qin D. Liu X. Li B. Li L. (2016b). Evaluation of current methodologies for rapid identification of methicillin-resistant *Staphylococcus aureus* Strains. *Basic Clin. Pharmacol.* 118:33.

[B44] Xu Z. Liang Y. Lin S. Chen D. Li B. Li L. (2016c). Crystal violet and XTT assays on *Staphylococcus aureus* biofilm quantification. *Curr. Microbiol.* 73 474–482. 10.1007/s00284-016-1081-1 27324342

[B45] Xu Z. Li L. Shirtliff M. E. Alam M. J. Yamasaki S. Shi L. (2009). Occurrence and characteristics of class 1 and 2 integrons in *Pseudomonas aeruginosa* isolates from patients in Southern China. *J. Clin. Microbiol.* 47 230–234. 10.1128/jcm.02027-08 19020065 PMC2620863

[B46] Xu Z. Li L. Shi L. Shirtliff M. E. (2011a). Class 1 integron in staphylococci. *Mol. Biol. Rep.* 38 5261–5279. 10.1007/s11033-011-0676-7 21258866 PMC3136644

[B47] Xu Z. Li L. Shirtliff M. E. Peters B. M. Li B. Peng Y. (2011b). Resistance class 1 integron in clinical methicillin-resistant *Staphylococcus aureus* strains in southern China, 2001-2006. *Clin. Microbiol. Infect.* 17 714–718. 10.1111/j.1469-0691.2010.03379.x 21521411

[B48] Xu Z. Li L. Zhao X. Chu J. Li B. Shi L. (2011c). Development and application of a novel multiplex polymerase chain reaction (PCR) assay for rapid detection of various types of staphylococci strains. *AFR. J. Microbiol. Res.* 5 1869–1873.

[B49] Xu Z. Li L. Shirtliff M. E. Peters B. M. Peng Y. Alam M. J. (2010). First report of class 2 integron in clinical *Enterococcus faecalis* and class 1 integron in *Enterococcus faecium* in South China. *Diagn. Micro. Infect. Dis.* 68 315–317. 10.1016/j.diagmicrobio.2010.05.014 20846812

[B50] Xu Z. Luo Y. Mao Y. Peng R. Chen J. Soteyome T. (2020). Spoilage lactic acid bacteria in the brewing industry. *J. Microbiol. Biotech.* 10.4014/jmb.1908.08069 [Epub ahead of print]. 31986245 PMC9728350

[B51] Xu Z. Li L. Alam M. J. Zhang L. Yamasaki S. Shi L. (2008a). First confirmation of integron-bearing methicillin-resistant *Staphylococcus aureus*. *Curr. Microbiol.* 57 264–268. 10.1007/s00284-008-9187-8 18594909

[B52] Xu Z. Shi L. Alam M. J. Li L. Yamasaki S. (2008b). Integron-bearing methicillin-resistant coagulase-negative staphylococci in South China, 2001-2004. *FEMS Microbiol. Lett.* 278 223–230. 10.1111/j.1574-6968.2007.00994.x 18096018

[B53] Xu Z. Shi L. Zhang C. Zhang L. Li X. Cao Y. (2007). Nosocomial infection caused by class 1 integron-carrying *Staphylococcus aureus* in a hospital in South China. *Clin. Microbiol. Infect.* 13 980–984. 10.1111/j.1469-0691.2007.01782.x 17803751

[B54] Xu Z. Xie J. Peters B. M. Li B. Li L. Yu G. (2017a). Longitudinal surveillance on antibiogram of important Gram-positive pathogens in Southern China, 2001 to 2015. *Microb. Pathogen.* 103 80–86. 10.1016/j.micpath.2016.11.013 27894963

[B55] Xu Z. Xu X. Qi D. Yang L. Li B. Li L. (2017b). Effect of aminoglycosides on the pathogenic characteristics of microbiology. *Microb. Pathogen.* 113 357–364. 10.1016/j.micpath.2017.08.053 28867624

[B56] Xu Z. Xie J. Yang L. Chen D. Peters B. M. Shirtliff M. E. (2018). Complete sequence of pCY-CTX, a plasmid carrying a phage-like region and an ISEcp1-Mediated Tn2 element from *Enterobacter cloacae*. *Microb. Drug Resist.* 24 307–313. 10.1089/mdr.2017.0146 28876168

[B57] Yaron S. Matthews K. R. (2002). A reverse transcriptase-polymerase chain reaction assay for detection of viable *Escherichia coli* O157:H7: investigation of species target genes. *J. Appl. Microbiol.* 92 633–640. 10.1046/j.1365-2672.2002.01563.x 11966903

[B58] You R. Gui Z. Xu Z. Shirtliff E. M. Yu G. Zhao X. (2012). Methicillin-resistance *Staphylococcus aureus* detection by an improved rapid PCR assay. *Afr. J. Microbiol. Res.* 6 7131–7133.

[B59] Zeinhom M. M. A. Wang Y. Song Y. Zhu M. Lin Y. Du D. (2018). A portable smart-phone device for rapid and sensitive detection of *E. coli* O157:H7 in Yoghurt and Egg. *Biosens. Bioelectron.* 99 479–485. 10.1016/j.bios.2017.08.002 28822314

[B60] Zhang S. Ye C. Lin H. Lv L. Yu X. (2015). UV disinfection induces a vbnc state in *Escherichia coli* and *Pseudomonas aeruginosa*. *Environ. Sci. Technol.* 49 1721–1728. 10.1021/es505211e 25584685

[B61] Zhao X. Li M. Xu Z. (2018a). Detection of foodborne pathogens by surface enhanced raman spectroscopy. *Front. Microbiol.* 9:1236 10.3389/fpubh.2014.001236 PMC6005832 29946307

[B62] Zhao X. Yu Z. Xu Z. (2018b). Study the features of 57 confirmed CRISPR Loci in 38 strains of *Staphylococcus aureus*. *Front. Microbiol.* 9:1591 10.3389/fpubh.2014.001591 PMC6070637 30093886

[B63] Zhao X. Lin C. Wang J. Oh D. H. (2014). Advances in rapid detection methods for foodborne pathogens. *J. Microbiol. Biotechnol.* 24 297–312. 24375418 10.4014/jmb.1310.10013

[B64] Zhao X. Li Y. Wang L. You L. Xu Z. Li L. (2010a). Development and application of a loop-mediated isothermal amplification method on rapid detection *Escherichia coli* O157 strains from food samples. *Mol. Biol. Rep.* 37 2183–2188. 10.1007/s11033-009-9700-6 19685165

[B65] Chu J. Li Y. Li Y. Xu Z. (2010b). Development and application of a rapid and simple loop-mediated isothermal amplification method for food-borne *Salmonella* detection. *Food Sci. Biotechnol.* 19 1655–1659. 10.1007/s10068-010-0234-4

[B66] Zhao X. Wang L. Chu J. Li Y. Li Y. Xu Z. (2010c). Rapid detection of vibrio parahaemolyticus strains and virulent factors by loop-mediated isothermal amplification assays. *Food Sci. Biotechnol.* 19 1191–1197. 10.1007/s10068-010-0170-3

[B67] Zhao X. Wang L. Li Y. Xu Z. Li L. He X. (2011). Development and application of a loop-mediated isothermal amplification method on rapid detection of *Pseudomonas aeruginosa* strains. *World J. Microb. Biot.* 27 181–184. 10.1007/s11274-010-0429-0

[B68] Zhong N. Gui Z. Xu L. Huang J. Hu K. Gao Y. (2013). Solvent-free enzymatic synthesis of 1, 3-diacylglycerols by direct esterification of glycerol with saturated fatty acids. *Lipids Health Dis.* 12:15. 10.1186/1476-511X-12-65 PMC3680111 23656739

